# High prevalence of chronic kidney disease in Iran: a large population-based study

**DOI:** 10.1186/1471-2458-9-44

**Published:** 2009-01-31

**Authors:** Farhad Hosseinpanah, Farshad Kasraei, Amir A Nassiri, Fereidoun Azizi

**Affiliations:** 1 Obesity Research Center, Research Institute for Endocrine Sciences, Shahid Beheshti University Medical Campus (M.C), Tehran, Iran; 2 Endocrine Research Center, Research Institute for Endocrine Sciences, Shahid Beheshti University Medical Campus (M.C), Tehran, Iran

## Abstract

**Background:**

Chronic kidney disease (CKD) is a global public health threat, associated with an alarming increase in morbidity and mortality. The importance is the worldwide increase in its incidence and prevalence.

**Methods:**

In this cross-sectional study, we estimate the prevalence and determine the associated factors of chronic kidney disease in a representative sample of 10063 participants aged over 20 years, in Tehran, Iran. Chronic kidney disease was defined as estimated glomerular filtration rate less than 60 mL/min/1.73 m2. Glomerular filtration rate was estimated from abbreviated prediction equation provided by the Modification of Diet in Renal Disease study (MDRD).

**Results:**

Overall prevalence of CKD with the abbreviated MDRD equation was 18.9% (95% confidence interval (CI) 18.2, 20.6). Age adjusted prevalence of CKD was 14.9% (95%CI 14.2, 15.6). Factors associated to CKD include age(years)(odds ratio(OR) 1.1, 95% CI 1.0 to 1.2), female gender (OR 3.1, 95% CI 2.6, 3.7), BMI (BMI 25 to <30 OR 1.5, 95% CI 1.3, 1.8 and BMI ≥ 30 OR 1.6, 95% CI 1.3, 2.0), high waist circumference (OR 1.2, 95% CI 1.1, 1.4), hypertension (OR 1.2, 95% CI 1.1, 1.4), and dyslipidemia (OR 1.3, 95% CI 1.1, 1.5).

**Conclusion:**

CKD with its high prevalence poses a definite health threat in Iran.

## Background

Chronic kidney disease (CKD) is a worldwide public health problem, associated with a considerable increase in morbidity and mortality. Of importance is its global increase in incidence and prevalence [1,2]. Outcomes of CKD include not only progression to kidney failure but also complications of reduced kidney function and increased risk of cardiovascular disease and all-cause mortality overall, although patients with CKD are far more likely to die, principally from cardiovascular disease, than to develop kidney failure [3-5]. However CKD is often undiagnosed and its complications are often untreated [6-8]. There is convincing evidence that CKD can be prevented or its progression delayed, if effective management is initiated in time. Hence, identifying patients with CKD and providing prompt intervention play an important role in appropriate management of CKD [9-11].

The prevalence of CKD has been addressed in several studies. In the United States, cross-sectional analysis of the most recent National Health and Nutrition Examination Surveys (NHANES) showed that the prevalence of CKD increased from 10.0% in 1988–1994 to 13.1% in 1999–2004 [2]. Studies from Europe, Australia, and Asia, also, confirm the high prevalence of CKD. The prevalence of reduced GFR in Australia was 11.2 percent [12]. Singapore, a south-east Asian country, reported a CKD prevalence of 10.1 percent, while the prevalence of CKD in Japanese general population was reported to be 18.7% [13,14]. However, much less information is available on prevalence rates elsewhere, from the developing regions of the Middle East in particular.

There is strong evidence that development and progression of CKD are outcomes of exposure to cardiovascular disease risk factors [15]. In many studies, the known risk factors for development and progression of CKD cited are age, diabetes mellitus (DM), hypertension, obesity, dyslipidemia and smoking [16-24].

To prevent and initiate appropriate management of CKD, it is crucial to have precise prevalence rates. The aim of this study was to estimate the prevalence and determine the associated factors of CKD in a large population based study in Tehran, Iran.

## Methods

The Tehran Lipid and Glucose Study (TLGS) is an ongoing population-based cohort study, with continuous recruitment since December 1997, which has been designed to determine risk factors for atherosclerosis in Tehran's urban population and to develop population based measures aimed at changing life styles and halting the increasing trend of DM and dyslipidemia [25]. The study is divided into two phases: a cross-sectional study of the prevalence of non-communicable diseases such as DM and cardiovascular disease and their associated risk factors, and a prospective 20-year follow up study. A multistage stratified cluster random sampling technique was used to select 15005 people aged ≥ 3 years from the urban district 13 of Tehran, the capital of the Islamic Republic of Iran. During sampling, the list of all households under coverage of the district's three healthcare centers (the official bodies responsible for vaccination programs and collection of health-related statistics in a district) was used; a random sample of the households, stratified by healthcare centre to achieve a distribution similar to the original population, was chosen; from each household, all members above the age of 3 years were recruited. The study began in December 1997 and the cross-sectional phase completed in 2000. District 13 is located in the centre of Tehran and the age distribution of its population is representative of the overall population of Tehran. Designated residents were sent an invitation requesting their participation [25]. The crude response rate in the TLGS participants was approximately 57.5%. The reason for no response have been investigated and there was no significant difference regarding age and sex between responders and nonresponders [26].

At the beginning of the cross-sectional phase, all participants provided written informed consent, which was approved by the institutional ethics committees (Research Institute for Endocrine Sciences) and was conducted in accordance with the principles of the Declaration of Helsinki. Thereafter, demographic data collection and anthropometric examinations were undertaken by trained general physicians. Weight was recorded using a Seca 707 weighing machine (range 0.1–150 kg) with an accuracy of up to 100 g. The precision of the machine was checked after every 10 measurements. Height was measured without shoes using a tape stadiometer with a minimum measurement of 1 mm. Body mass index (BMI) was calculated by dividing weight (in kg) by height squared (in m^2^). Systolic and diastolic blood pressures were measured using a standardized mercury sphygmomanometer on the right arm after a 15-min rest in the supine position. Blood samples were drawn between 08.00 and 09.00 h into vacutainer tubes after a 12–14-h overnight fast according to the standardized protocol of the TLGS. Then subjects underwent a standardized 75-g oral glucose tolerance test (OGTT). All samples were centrifuged within 35–40 min of collection (2500 r.p.m. (1000 g), 30 min and 4°C). All blood biochemical analyses were performed at the TLGS research laboratory on the day of blood collection. Analyses were performed using the Selectra 2^® ^autoanalyser (Vital Scientific, Spankeren, the Netherlands). Plasma glucose was assayed using the glucose oxidase method [Pars Azmon Inc., Iran; with inter- and intra-assay coefficients of variation (CVs) of 3% and 0.8%, respectively]. For 2 h-OGTT, 75 g glucose was administrated orally and plasma glucose was measured 2 hours later (2 h-PG) [27].

Plasma total cholesterol(TC) and triglyceride(TG) levels were measured using enzymatic colorimetric kits (Pars Azmon Inc.; inter- and intraassay CVs were 2 and 0.5% for TC and 1.6 and 0.6% for TGs, respectively). Sera creatinine levels were measured according to the standard colorimetric Jaffe_Kinetic reaction method (Pars Azmon Inc., Iran; with inter- and intra-assay CVs of 2.5% and 1.9%, respectively, and sensitivity of 0.2 mg/dl). The assay range was 18–1330 μmol (0.2–15 mg/dl). Reference intervals according to producer recommendation were: 53–97 μmol (0.6–1.1 mg/dl) and 80–115 μmol (0.9–1.3 mg/dl) in women and men respectively in serum/plasma. Assay performance was monitored after every 25 tests using control serum, Precinorm (cat. no. 1446070; Boehringer Mannheim, Germany) for normal range and Precipath (cat. no. 171778; Boehringer Mannheim) for pathological ranges [25].

From 15005 participants of the cross-sectional phase of TLGS, we excluded subjects <20 years of age. Data of 10368 eligible individuals were gathered and 305 individuals, who had missing data for calculation of creatinine, were excluded. Analysis was conducted on the data of the remaining 10063 participants.

### Definitions

According to the Kidney Disease Outcome Quality Initiative (K/DOQI) guideline, chronic kidney disease is defined as either kidney damage or Glomerular Filtration Rate (GFR) <60 mL/min/1.73 m^2 ^(1.0 mL/s/1.73 m^2^) for >3 months. Kidney damage is defined as pathologic abnormalities or markers of damage, including abnormalities in blood or urine tests or imaging studies [28]. For this study GFR was estimated from abbreviated prediction equation provided by the Modification of Diet in Renal Disease (MDRD) study as following:

Abbreviated MDRD study equation:

*GFR *= 186 × (*SCr*)^-1.154 ^× (Age)-^0.203 ^× (0.742 if female) × (1.210 if African-American)

In this equation, GFR is expressed as mL/min per 1.73 m^2^, and serum creatinine (Scr) is expressed as mg/dL [29].

The classification of CKD by stages was done also according to the Kidney Disease Outcome Quality Initiative (K/DOQI) criteria as below:

#### Stage 1

Normal GFR (greater than 90 ml/min per 1.73 m^2 ^(1.50 mL/s/1.73 m^2^)) and persistent albuminuria.

#### Stage 2

A GFR between 60 to 89 ml/min per 1.73 m^2 ^(1.00 to 1.49 mL/s/1.73 m^2^) and persistent albuminuria.

#### Stage 3

A GFR between 30 and 59 ml/min per 1.73 m^2 ^(0.50 to 0.99 mL/s/1.73 m^2^).

#### Stage 4

A GFR between 30 and 15 ml/min per 1.73 m^2 ^(0.25 to 0.49 mL/s/1.73 m^2^).

#### Stage 5

A GFR less than 15 ml/min per 1.73 m^2 ^(0.25 mL/s/1.73 m^2^) or end-stage renal disease [28].

BMI (Body mass index) was categorized as 3 groups of <25 kg/m^2^, 25 to <30 kg/m^2 ^(overweight), and ≥ 30 kg/m^2 ^(obese). Abnormal waist circumference was set as ≥ 102 cm in men, and ≥ 88 cm in women according to the Third Report of the National Cholesterol Education Program (NCEP) Expert Panel on Detection, Evaluation, and Treatment of High Blood Cholesterol in Adults (Adult Treatment Panel III) [30]. Hypertension was defined according to the Seventh Report of the Joint National Committee (JNC7) on Prevention, Detection, Evaluation, and Treatment of High Blood Pressure criteria as systolic blood pressure ≥ 140 mmHg or diastolic blood pressures ≥ 90 mmHg, or patients taking antihypertensive agents [31]. Dyslipidemia was defined according to the Executive Summary of the Third Report of the National Cholesterol Education Program (NCEP) Expert Panel on Detection, Evaluation, and Treatment of High Blood Cholesterol in Adults (Adult Treatment Panel III) as serum triglyceride of ≥ 200 mg/dl or cholesterol of ≥ 240 mg/dl and also included subjects taking lipid lowering medications [30]. Smoking was defined as participants who smoked cigarettes daily or occasionally or participants who smoked before. DM (diabetes mellitus) defined according to the criteria of the American Diabetes Association (ADA) as fasting plasma glucose ≥ 126 mg/dl or 2-h postload ≥ 200 mg/dl, included patients who were known cases of diabetes and use insulin or oral glucose-lowering agents [27].

### Statistical Methods

All continuous data with normal distribution are expressed as mean ± SD and skewed parameters as median, interquartile 25–75% (IQ_25–75_), and categorical variables are expressed as percentage. Age adjusted prevalence was estimated with the reference population group of Iran according to the data from the 1996 census. Difference for continuous variables was assessed by using the *t*-test, whereas difference for categorical variables was assessed with the *Chi Square Test*. A multivariate logistic regression model was used to estimate the odds ratio (OR) of related factors with CKD. Age (years), sex (reference: male), BMI (reference: < 25 kg/m^2^), abnormal waist circumference, hypertension, dyslipidemia, DM and smoking in dichotomous fashions were considered as independent variables. All statistical analyses were performed by SPSS software (version 15.0). Differences with probability values < 0.05 were considered statistically significant.

## Results

**Figure 1:**
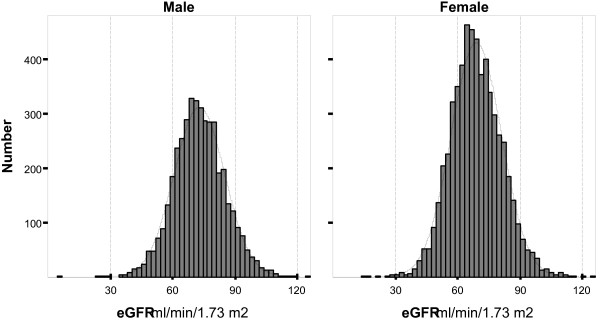
**GFR distribution**. Histograms demonstrating GFR distribution for male and female participants of the Tehran Lipid and Glucose Study (TLGS) aged 20 years and over.

The baseline characteristics of the study population are listed in Table 1. A total of 10063 participants aged ≥ 20 years were analyzed; while population age ranged between 20 and 90 years, the participants were mostly young and the mean age was 42.7 ± 14.9 years (median and IQ_25–75 _41 and 31–54 years, respectively). Of the total study population, 41.9% (n = 4223) were male. The mean (± SD) of height for men and women were 169.7 (± 6.8) and 156.5 (± 6.1) respectively and the mean (± SD) of weight for men and women were 74.3 (± 12.7) and 67.3 (± 12.4) respectively. Normal BMI was detected in 36.7% (n = 3614) of participants, the other 63.3% (n = 6232) were overweight or obese; from the aspect of abdominal obesity, 33.3% (n = 3349) had abnormal waist circumference, which was more common in women (48.9%, n = 2855) than in men (11.7%, n = 494). The prevalence of DM, hypertension and dyslipidemia was 13.7%, 25.8% and 44.3%, respectively. In our population, 13.1% were smokers. The mean (± SD) creatinine and eGFR were 1.1 (± 0.2) mg/dl (97.2 μmol/L), and 70.6 (± 12.3) mL/min/1.73 m^2 ^(1.17 mL/s/1.73 m^2^), respectively with minimum and maximum of 0.5 mg/dl (44.2 μmol/L) and 9.2 mg/dl (813.2 μmol/L) for creatinine and, 5.9 mL/min/1.73 m^2 ^(0.09 mL/s/1.73 m^2^) and 126 mL/min/1.73 m^2 ^(2.10 mL/s/1.73 m^2^) for eGFR, while the mean (± SD) of serum creatinine and eGFR for men were 1.2 (± 0.2) mg/dl and 72.9 (± 12.2) mL/min/1.73 m^2 ^respectively and those of serum creatinine and eGFR for women were 1.0 (± 0.1) mg/dl and 69.0 (± 12.2) mL/min/1.73 m^2 ^(Figure 1).

**Table 1:** Baseline characteristics of 10 063 participants of the Tehran Lipid and Glucose Study (TLGS) aged 20 years and over.

	n	%
Gender (male)	4223	41.9
Age(years)		

20–39	4782	47.6

40–59	3510	34.8

60–69	1325	13.2

≥ 70	446	4.4

Body mass index (kg/m^2^)		

≤ 25	3614	36.7

25–30	3951	40.1

≥ 30	2281	23.2

Abnormal waist circumference in men	494	11.7

Abnormal waist circumference in women	2855	48.9

Diabetes mellitus	1381	13.7

Hypertension	2593	25.8

Dyslipidemia	4462	44.3

Smoking	1300	13.1

**Figure 2:**
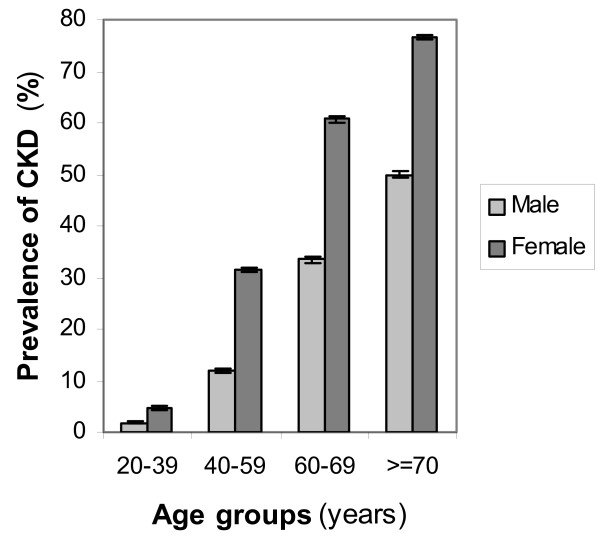
**Age-specific prevalence of CKD**. Prevalence of CKD for each age group by gender in 10,063 participants of the Tehran Lipid and Glucose Study (TLGS) aged 20 years and over is shown. Error bars represent standard error with 95% confidence interval.

Overall prevalence of CKD, based on eGFR calculated with the abbreviated MDRD equation was 18.9% (95% CI 18.2, 20.6); while age adjusted prevalence of CKD was 14.9% (95%CI 14.2, 15.6). From participants who had CKD, 99.2% were in the stage 3 of CKD, 0.7% (n = 13) in the stage 4 and only 0.1% (n = 2) in the stage 5 of CKD (Table 2). The prevalence of CKD increased with increasing age, therefore the prevalence of CKD was highest among people within the age group of ≥ 70 years. In each age group the prevalence of CKD in women was higher than men (Figure 2).

**Table 2:** Prevalence of CKD among different age groups in 10 063 participants of the Tehran Lipid and Glucose Study (TLGS) aged 20 years and over.

Age groups	n	CKD%	*95% CI*
**Men**			

20–39	1897	1.8	1.2–2.4

40–59	1413	12.0	11.1–12.9

60–69	651	33.5	29.9–37.1

≥ 70	262	50.0	44.0–56.0

**Total**	**4223**	**13.1**	**12.1–14.1**

**Women**			

20–39	2885	4.6	3.8–5.4

40–59	2097	31.5	29.5–33.5

60–69	674	60.8	57.1–64.5

≥ 70	184	76.6	70.5–82.7

**Total**	**5840**	**23.0**	**21.9–24.1**

**All**			

20–39	4782	3.5	3.0–4.0

40–59	3510	23.6	22.2–25.0

60–69	1325	47.4	44.7–56.5

≥ 70	446	61.0	56.5–65.5

**Total**	**10063**	**18.9**	**18.1–19.7**

CI, confidence interval

In bivariate analysis, the factors significantly associated with CKD other than older age were being female gender, obesity, having diabetes mellitus (DM), being hypertensive and having dyslipidemia, but nonsmoking was associated with CKD (Table 3). Using multiple logistic regression analysis, the multivariate-adjusted ORs for presence of CKD were significant for age (OR 1.1, 95% CI 1.0, 1.2), female gender (OR 3.1, 95% CI 2.6, 3.7), BMI (BMI 25 to <30 OR 1.5, 95% CI 1.3, 1.8 and BMI ≥ 30 OR 1.6, 95% CI 1.3, 2.0), abnormal waist circumference (OR 1.2, 95% CI 1.1, 1.4), hypertension (OR 1.2, 95% CI 1.1, 1.4), and dyslipidemia (OR 1.3, 95% CI 1.1, 1.5 (Table 4).

**Table 3:** CKD associated factors in individuals with and without CKD in 10 063 participants of the Tehran Lipid and Glucose Study (TLGS) aged 20 years and over.

variable		CKD n = 1897	Without CKD n = 8166	P value
Sex	male	553(29.2)	3670(44.9)	< 0.001

	female	1344(70.8)	4496(55.1)	

				

BMI(kg/m^2^**)**	≤ 25	392(21.0)	3222(40.4)	

	25 to 30	852(45.6)	3099(38.8)	

	≥ 30	625(33.4)	1656(20.8)	<0.001

				

Waist circumference	abnormal	1049(55.3)	2300(28.2)	<0.001

				

Diabetes	diabetic	494(26.0)	887(10.9)	<0.001

				

Hypertension	hypertensive	982(51.8)	1611(19.7)	< 0.001

				

Dyslipidemia	dyslipidemic	1244(65.6)	3218(39.4)	< 0.001

				

Smoking	smoker	140(7.4)	1160(14.4)	<0.001

Values are numbers (percentages); BMI, body mass index

**Table 4:** Multivariate logistic regression for factors related to CKD in 10 063 participants of the Tehran Lipid and Glucose Study (TLGS) aged 20 years and over.

Variable	CKD (n = 1897)	Total (n = 10063)	Adjusted odds ratio	95%CI	P
Age(years)			1.1	1.1–1.2	<0.001

Sex					

male	553(29.2)	4223(42.0)	1.0		<0.001

female	1344(70.8)	5840(58.0)	3.1	2.6–3.7	

BMI(kg/m^2^)					

<25	392(21.0)	3614(36.7)	1.0		

25 to <30	852(45.6)	3951(40.1)	1.5	1.3–1.8	<0.001

≥ 30	625(33.4)	2281(23.2)	1.6	1.3–2.0	<0.001

Waist circumference					

Normal	848(44.7)	6714(66.7)	1.0		0.002

Abnormal	1049(55.3)	3349(33.3)	1.2	1.1–1.4	

Blood pressure					

Normotensive	915(48.2)	7470(74.2)	1.0		0.004

Hypertensive	982(51.8)	2593(25.8)	1.2	1.1–1.4	

Dyslipidemia					

Normolipidemic	653(34.4)	5601(55.7)	1.0		<0.001

Dyslipidemic	1244(65.6)	4462(44.3)	1.3	1.1–1.5	

Diabetes					

Nondiabetic	1403(74.0)	8682(86.3)	1.0		0.6

Diabetic	494(26.0)	1381(13.7)	0.9	0.8–1.1	

Smoking					

Nonsmoker	1741(92.6)	8609(86.9)	1.0		0.778

Smoker	140(7.4)	1300(13.1)	1.1	0.9–1.3	

BMI, body mass index; CI, confidence interval

## Discussion

This study shows a remarkably high prevalence of chronic kidney disease (CKD) among individuals, aged 20 years and above, in an urban population in Iran. The age adjusted prevalence of CKD, stages 3 to 5, in this population based study in Iran is 14.9% (95% CI 14.2,15.5), and associated factors of CKD include older age, female gender, increased BMI and abnormal waist circumference, hypertension, and dyslipidemia.

The prevalence of CKD stages 3 to 5, as addressed in several studies, differs in different countries and ethnic groups worldwide. The prevalence of overall CKD in the adult US population according to the third national health and nutrition examination survey (NHANESIII) was 11%, but the prevalence of CKD stages 3 to 5 was 4.7% [32]. The prevalence of CKD stages 1 to 4 in Norway, estimated by MDRD study equation was 11.2%, but the prevalence of CKD stages 3 and 4 was 4.6% [33]. In the Chinese general population aged 35 to 74 years, the prevalences of CKD stage 3 and stage 4, estimated by the MDRD study equation, were 2.4% and 0.14%, respectively which are far lower than those of our results [34]. Not too many studies have reported results similar to ours. The prevalence of CKD stages 3 to 5 in the Japanese general population predicted by the MDRD equation modified by a Japanese coefficient was about 20% [13]. The prevalence of CKD stages 3 and 4 in Thailand estimated recently using the simplified MDRD equation was 13.8%, and the overall prevalence of CKD in a study in Pakistan with a small sample size was 29.9% [35,36].

The prevalence of CKD stages 3 to 5 in our study is noticeably more than that reported from developed countries. There might be some probable reasons for this discrepancy. First, the risk factors of CKD including diabetes and dyslipidemia in our population were more than in other countries, although the prevalence of hypertension and abnormal BMI were comparable to other studies [2,13,37]. Second, the MDRD study formula was developed based on 1628 participants in whom GFR was measured with a reference method, whereas these patients had CKD (mean GFR of 39.8 mL/min/1.73 m^2^). This is important because the relationship between serum creatinine and GFR differs in healthy and CKD populations and the MDRD formula is most accurate for the GFR<60 mL/min/1.73 m^2^, therefore the MDRD study formula systematically underestimates GFR in healthy populations [38]. Furthermore it is possible that definition of CKD based on calculation using MDRD formula is not reliable in Asian an Iranian populations because this formula has not been validated in these populations. This may lead to the prevalence of CKD being overestimated in our study.

Many studies have evaluated various associated factors of CKD, and in the present study there is a significant association between each of the factors; age, female gender, BMI, hypertension and CKD similar to the previous studies [19,39,40]. Although age and gender were included in the MDRD equation, they were significant as independent associated risk factors for CKD in our analysis. The higher prevalence of CKD in female gender might be caused by lower physical activity, and higher prevalence of cardio metabolic risk factors in Iranian females. And the increasing prevalence of decreased kidney function in older individuals might result from an increase in age-related risk factors for the development of CKD. While diabetes in most studies is mentioned as an independent predictor for CKD, in our study despite a high prevalence of diabetes in our population, in the multivariate regression model it was not significantly associated with CKD. A probable explanation for this might be the recent development of diabetes in our population, i.e. over than 70% of cases were newly diagnosed. Another unexpected finding in our study was that, in contrast to other studies, nonsmoking was associated with CKD, although in the multivariate analysis it did not remain in the model. This can be explained by our low prevalence of smoking in comparison to the other studies and also our inability to take into account dose response relationship between smoking and CKD. In addition, over two thirds of CKD cases were females (70.8%), among whom there was no significant difference between smoker and nonsmoker percentages(3% versus 3.6%, P value = 0.32); whereas most of smokers were males, they comprised less than one third of CKD cases(29.2%). In the present study, income and educational levels were the most important determinants of socio-economic status of participants; however because of the homogeneity of the geographical location of participants' residential area, which is a reliable indicator of their equal economic level; we considered their education as the socio-economic criterion. From this aspect 64% of the participants had the educational level of high school diploma and above and the relationship between CKD and under diploma was significant (data not shown).

Our survey has both strengths and limitations. We surveyed a large number of subjects during a population-based study; however the limitations of our analysis definitely deserve comment. Data regarding urinary albumin and protein excretion were not collected, and hence the prevalence of stages I and II CKD could not be estimated in this population. Another limitation is that we accepted participants to have CKD just with a single creatinine measurement but we can not ensure that all identified subjects with CKD had persistently impaired renal function for at least 3 months. Although single creatinine measurement is considered appropriate for epidemiologic studies, by conducting only a one time screening, we may have overestimated the CKD prevalence in Iran. Furthermore, we did not calibrate our serum creatinine measurements to the Cleveland Clinic, where the Modification of Diet in Renal Disease (MDRD) eGFR equation was derived; nor did we validate the MDRD eGFR equation in a local population, and this could also cause an overestimation in the prevalence of CKD. Finally, the cross-sectional design of the present study makes it impossible to infer a causal relationship between CKD and associated factors.

## Conclusion

In summary, this high prevalence of chronic kidney disease (CKD) in Iran has obvious implications for the health of its citizens and for the appropriate allocation of health-care resources. CKD has become an important health problem associated with an alarming increase in morbidity and mortality and with decreased quality of life, although it can be prevented or its progression be delayed; hence identifying the patients with CKD and providing appropriate management is vital.

## Abbreviations

CKD: chronic kidney disease; GFR: glomerular filtration rate; eGFR: estimated glomerular filtration rate; MDRD: Modification of Diet in Renal Disease; ESRD: end stage renal disease; BMI: body mass index; DM: diabetes mellitus; CI: confidence interval; OR: odds ratio; IQ_25–75_: interquartile 25–75%; CV: coefficients of variation; OGTT: oral glucose tolerance test; TLGS: Tehran Lipid and Glucose Study; TC: total cholesterol; TG: triglyceride.

## Competing interests

The authors declare that they have no competing interests.

## Authors' contributions

FH contributed to design of the study, manuscript preparation, and reading and approval of the final manuscript. FK contributed to design of the study, analysis of data, writing of the manuscript and its revisions, and reading and approval of the final manuscript. AAN contributed to design of the study, analysis of data, revision of the manuscript, and reading and approval of the final manuscript. FA contributed to provision of data, and reading and approval of the final manuscript.

## Pre-publication history

The pre-publication history for this paper can be accessed here:


